# Ultra-High Molecular Weight Polyethylene (marPOR) is a Suitable Material for the Reconstruction of Orbital Floor Fracture Defects in Human Cadavers

**DOI:** 10.1007/s12663-022-01789-0

**Published:** 2022-09-28

**Authors:** Yannick Foerster, Marco Kesting, Frank Reinauer, Adem Aksu, Raimund Preidl

**Affiliations:** 1 Department of Oral and Maxillofacial Surgery, University of Erlangen-Nuremberg, Erlangen, Germany; 2 Department of Oral and Maxillofacial Surgery, University of Erlangen-Nuremberg, Erlangen, Germany; 3 KLS Martin Group, Tuttlingen, Germany; 4 Department of Oral and Maxillofacial Surgery, University of Erlangen-Nuremberg, Erlangen, Germany

**Keywords:** Orbital floor fracture, Ultra-high molecular weight polyethylene, UHMWPE, marPOR, PDS, Ethisorb

## Abstract

**Purpose:**

Despite there being different materials for orbital floor reconstruction available today, outcomes are still not satisfying. In recent years, ultra-high molecular weight polyethylene (UHMWPE) has gained popularity in the field of orthopedic surgery due to its good biocompatibility and low infection rate. With its three-dimensional compound structure, it combines high stability and ductility, making it a potential material to be used for orbital floor reconstruction.

**Methods:**

In a cadaver study, an overall of eighteen orbits were included. Fractures of the inferior wall were induced and then reconstructed using Polyglactin 910/PDS composite (Ethisorb) and UHMWPE (marPOR). Orbits were scanned by cone-beam CT in each condition: Intact, fractured and reconstructed with Ethisorb, marPOR 0.85 mm and marPOR 1.5 mm. Segmented orbital volumes were calculated by specialized software (Disior bonelogic CMF).

**Results:**

All materials led to sufficient reconstruction of the initial orbital volumes (Ethisorb: *p* < *0.001*; marPOR 0.85 mm: *p* = *0.003*; marPOR 1.5 mm: *p* < *0.001*). Orbits that were reconstructed with marPOR 0.85 mm showed the least mean volume difference from intact orbital volumes.

**Conclusion:**

UHMWPE (marPOR) offers reliable reconstruction of orbital floor fractures combined with good stability, ductility and biocompatibility.

## Introduction

Orbital floor fractures are among the most frequent fractures of the midface [1]. Due to its delicate anatomical structures, the orbit is very susceptible to volume alterations caused by trauma. The most common blow-out-fracture enlarges the orbital volume, which in turn can lead to severe functional impairment, such as diplopia, enophthalmos, limitation of ocular movement and even vision loss [2]. Immediate surgical intervention to preserve the integrity of the orbit is therefore often necessary. In this context, restoration of preoperative orbital volume has proven to be the most useful numeric parameter for evaluating sufficient orbital reconstruction [3–5]. If there is no preoperative volume available, mirroring of the opposite orbit can also be used as reference [6]. Until today, polydioxanone (PDS) sheets are most commonly used for orbital floor reconstruction, followed by polyglactin 910/PDS composites and, for larger defects, titanium meshes [7]. However, the results are still not satisfying and around 20% of patients suffer from postoperative complications [8, 9]. Because of its high flexibility, PDS sheets and polyglactin 910/PDS composites gently adapt to the bony contour of the orbit, but on the other hand this leads to limited stability and higher rates of persisting enophthalmos and diplopia [7]. Therefore, both materials should be used only for small-to-moderate orbital floor fracture defects up to a maximum size of 1–2 cm^2^ (≙Jaquiéry I) [10]. Moreover, a significant number of patients with polyglactin 910/PDS implants experience postoperative infections [7, 8]. For larger defects, titanium mesh is widely used due to its stability and low distortion rate [11]. Despite titanium mesh, especially if pre-bent, offering reliable reconstruction of the orbital volume, fibrous reaction between the orbit and the mesh may prove devastating for the patient, causing cicatricial eye movement restriction and lid retraction [12, 13]. Beyond these serious complications, titanium implants can lead to tenderness, weather sensations and beam hardening in future x-ray examinations [14]. Therefore, the demand for a material that combines reliable orbital reconstruction, low infection rates and a flat, inert profile, rather than a mesh to prevent adhesions through the mesh, is unabated.

In recent years, ultra-high molecular weight polyethylene (UHMWPE) has become a popular material in the field of orthopedic surgery with excellent outcomes and low infection rates [15, 16]. It is radiolucent and cannot be imaged using x-rays. With its three-dimensional, highly porous compound structure, UHMWPE offers high stability combined with low dead weight. These properties make UHMWPE a suitable material to be used for orbital floor reconstruction. The aim of the present study is to investigate if UHMWPE is an equal material in terms of restoring preoperative orbital volumes in cadavers compared to a conventional polyglactin 910/PDS composite.

## Material and Methods

**Fig. 1:**
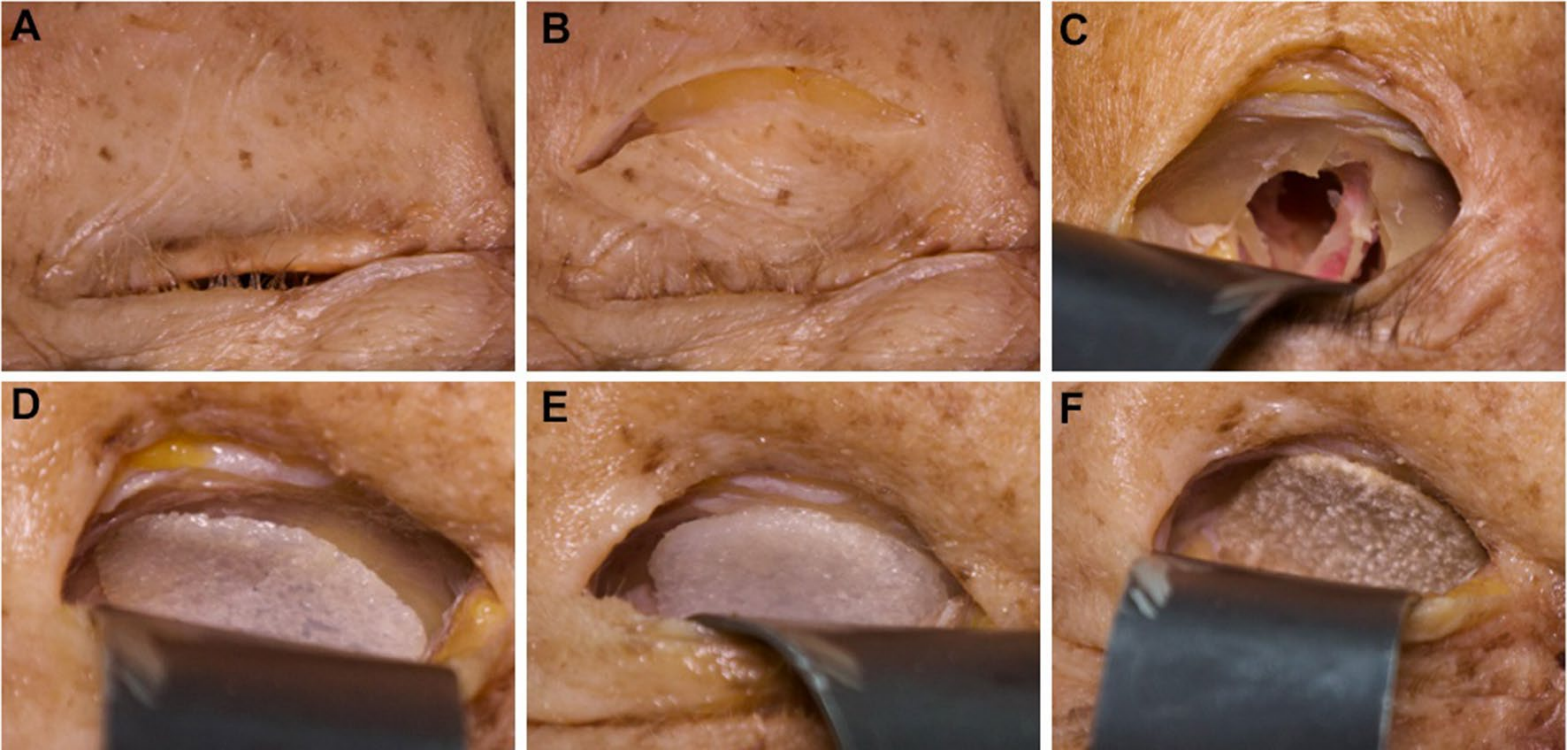
Surgical procedure. View on the left eye from overhead (**a**). Infraorbital incision was performed (**b**) and fatty tissue was dissected. After incision of the periosteum on the inferior margo and subperiostal preparation into the orbit, the orbital floor was exposed and then fractured by using a sharp raspatory (**c**). Orbital floor defects were then reconstructed with different materials: marPOR with a strength of 0.85 mm (**d**), marPOR with a strength of 1.5 mm (**e**) and Ethisorb (**f**)

The present study investigated an overall of eighteen orbits from nine human cadavers that were bequeathed to the Department of Anatomy, University of Erlangen-Nuremberg, Germany between 2018 and 2020. After cone-beam computed tomography (CBCT) images were taken from each cadaver head, inferior orbital walls were exposed via infraorbital approach and subperiosteal preparation. Thereafter, bilateral isolated trap-door fractures (≙Jaquiéry I) of the orbital floor were induced in a standardized manner using a raspatory (Fig. 1a–c), and all heads were rescanned. After reposition of herniated orbital tissue, UHMWPE implants (marPOR, KLS Martin Group, Tuttlingen, Germany) with a strength of 0.85 mm (Fig. 1d), 1.5 mm (Fig. 1e) and a Polyglactin 910/PDS composite sheet (Ethisorb, Ethicon, Johnson & Johnson, New Brunswick, NJ, USA) (Fig. 1f) were placed on the inferior orbital wall. All orbits were consecutively reconstructed with three different materials and rescanned in each case: marPOR 0.85 mm (Fig. 1d), marPOR 1.5 mm (Fig. 1e), Ethisorb (Fig. 1f).

**Fig. 2:**
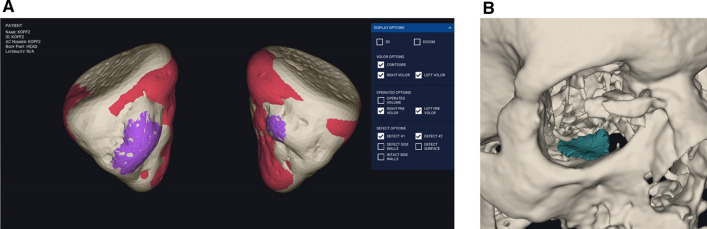
Segmented 3D model of the orbital volumes and fracture area. Images in digital imaging and communications in medicine (DICOM) format were transferred into the CMF bonelogic orbital software (Disior, Helsinki, Finland). The program automatically generates a segmented 3D model of the orbit (**a**; indicated in white, contours in red) and turns into numerical data. Moreover, volumes of the fracture (**a**; indicated in purple), the fracture area (**b**; indicated in green) and the maximum prolapse into the maxillary sinus (not indicated) are automatically analyzed

The intact, fractured (not reconstructed) and reconstructed orbital volumes (mL) were calculated automatically by using Disior Bonelogic CMF Orbital Software Version 2.1.30 (Disior, Helsinki, Finland). After importing images in digital imaging and communications in medicine (DICOM) format into the software, a segmented 3D model of the orbit was automatically created and turned into numerical data (Fig. 2a). Also, the fractured area (cm^2^) (Fig. 2b) and the maximum collapse (mm) were computed. Volume differences from intact orbital volumes were calculated for each case (not reconstructed, reconstructed with Ethisorb, marPOR 0.85 mm and marPOR 1.5 mm) and means were compared using the unpaired *t*-test (SPSS Version 28.0, SPSS Inc., Chicago, IL, USA). Data is expressed as box-and-whisker diagram, indicating minimum, maximum, median, first quartile and third quartile. A *p-value* lower than 0.05 was considered statistically significant.

## Results

**Fig. 3:**
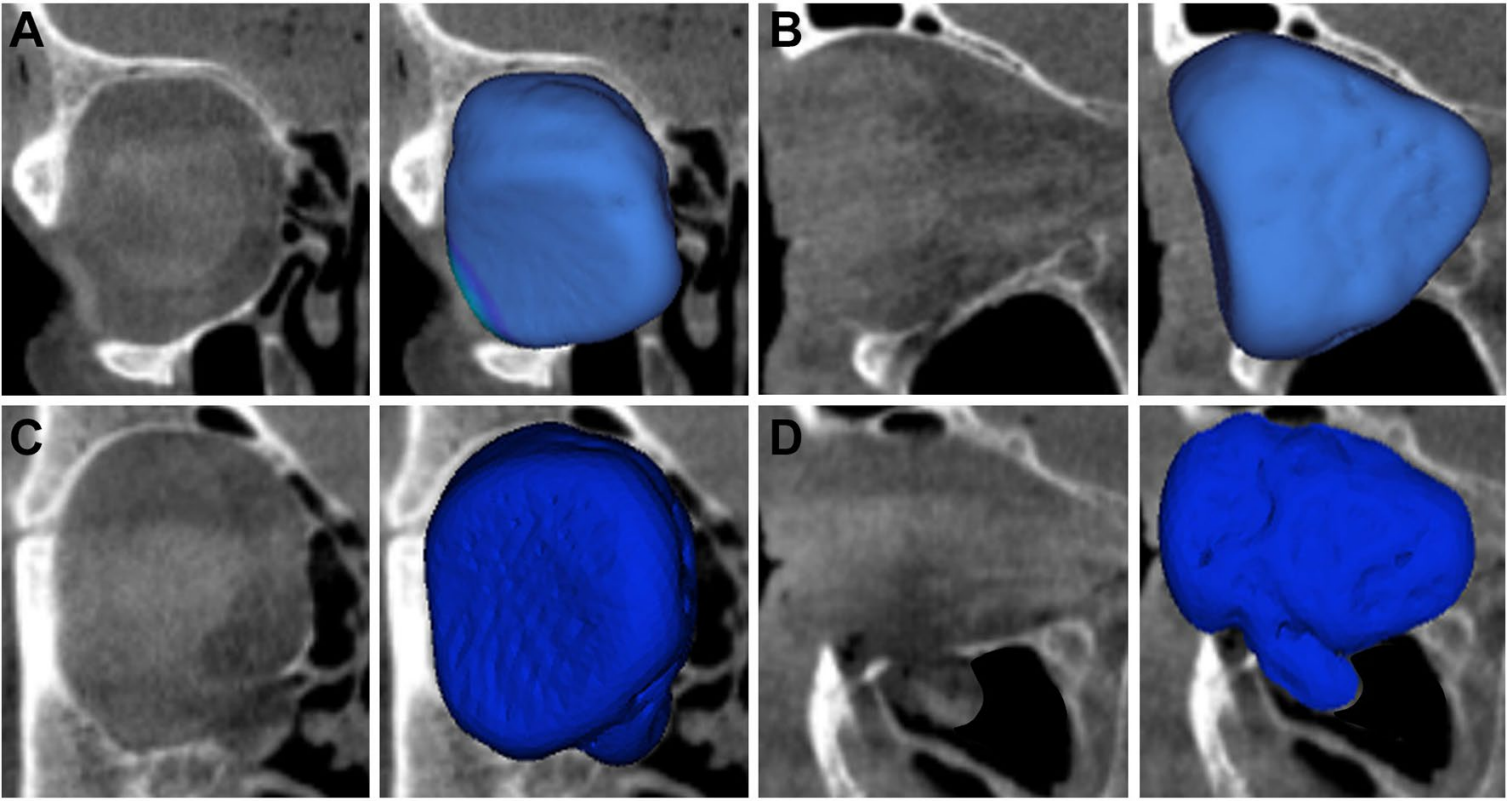
Intact and fractured orbits. Cone-beam CT scans of the right orbit in coronal (**a**, **c**) and sagittal (**b**, **d**) view. Segmented volumes of intact (light blue) and fractured orbits (dark blue) were automatically created by specialized software

**Fig. 4:**
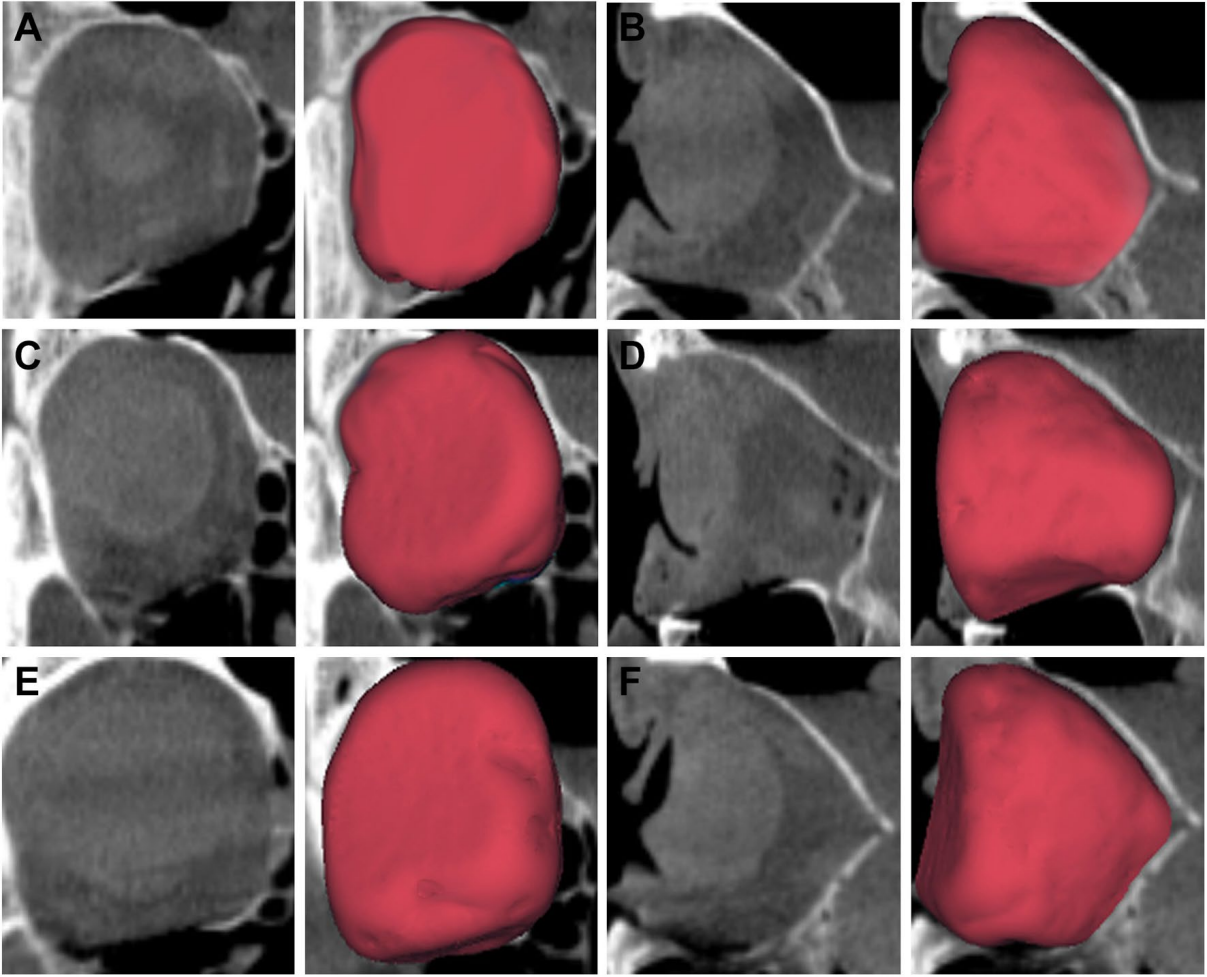
Reconstructed orbits. Cone-beam CT scans of the right orbit in coronal (**a**, **c**, **d**) and sagittal (**b**, **d**, **e**) view. Each orbit was reconstructed using Ethisorb (**a**, **b**), marPOR with a strength of 0.85 mm (**c**, **d**) and marPOR with a strength of 1.5 mm (**e**, **f**). Segmented volumes (in red) were automatically created by specialized software

The mean absolute volume ± standard deviation (SD) of the intact orbits was 29.84 ± 3.27 mL (Fig. 3a, b). The mean volume ± SD of the fractured (not reconstructed) orbits was 30.68 ± 3.24 mL (Fig. 3c, d). All materials used for orbital reconstruction reduced absolute orbital volumes ± SD: 29.63 ± 3.63 mL (Ethisorb) (Fig. 4a, b), 29.79 ± 3.78 mL (marPOR 0.85 mm) (Fig. 4c, d), 29.5 ± 3.49 mL (marPOR 1.5 mm) (Fig. 4e, f).

The mean absolute volume difference ± SD of the fractured (not reconstructed) orbit compared to the intact orbit (volume of fractured orbit – volume of intact orbit) was + 0.84 ± 0.71 mL. All investigated orbits exhibited higher volumes after the inferior wall was fractured (*p* < 0.001).

**Fig. 5:**
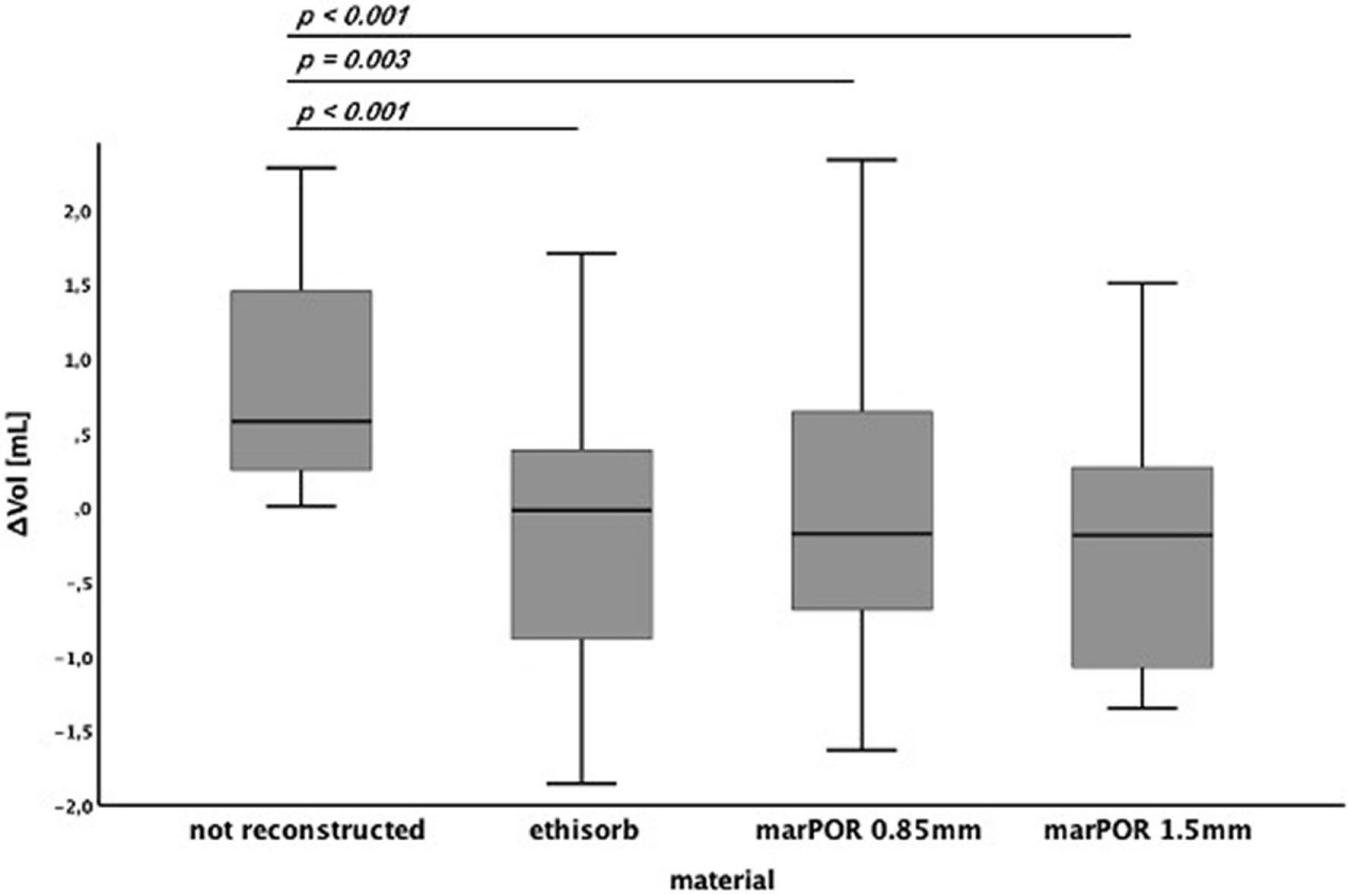
Absolute volume differences from intact orbital volumes. Each box indicates minimum, maximum, median, first quartile and third quartile. All materials significantly reduced mean volume differences from the mean volume of fractured (not reconstructed) orbits: Ethisorb: *p* < 0.001; marPOR 0.85 mm: *p* = 0.003; marPOR 1.5 mm *p* < 0.001

Compared to the fractured (not reconstructed) orbits, all reconstructed orbits showed significantly reduced absolute volume differences independent from the material that was used for orbital floor reconstruction (Ethisorb: *p* < 0.001; marPOR 0.85 mm: *p* = 0.003; marPOR 1.5 mm *p* < 0.001) (Fig. 5). Orbits that were reconstructed with marPOR 0.85 showed the least mean absolute volume difference ± SD (− 0.05 ± 0.95 mL) compared to Ethisorb (− 0.21 ± 0.93 mL) and marPOR 1.5 mm (− 0.34 ± 1.14 mL). All materials tended to overcompensate volume restoration, resulting in a smaller absolute mean volume compared to mean volume of intact orbits. However, these differences were not statistically significant.

The mean value ± SD of the fractured area was 1.54 ± 0.56 cm^2^. The mean maximal collapse ± SD was 3.74 ± 0.69 mm. All results are presented in Table 1.

**Table 1:** Results. Mean values ± standard deviation (SD)

	Intact	Fractured [not reconstructed]	Ethisorb	marPOR 0.85 mm	marPOR 1.5 mm
Absolute volume (mL)	29.84 ± 3.27	30.68 ± 3.24	29.63 ± 3.63	29.79 ± 3.78	29.5 ± 3.49
Absolute volume difference from intact orbital volume (mL)	–	+ 0.84 ± 0.71	− 0.21 ± 0.93	− 0.05 ± 0.95	− 0.34 ± 1.14
Fracture area (cm^2^)	–	1.54 ± 0.56	–	–	–
Max. collapse (mm)	–	3.74 ± 0.69	–	–	–

## Discussion

Although advances in biotechnology continue to introduce new materials for reconstruction of orbital floor defects, there is still inconclusive data about which material is best fit for orbital floor reconstruction [7]. However, current studies report PDS and Polyglactin 910/PDS composites, two materials which are widely used for orbital floor reconstruction, to be associated with increased infection rates and reduced ocular motility after surgery [7, 17, 18]. Due to their low stability, PDS and Polyglactin/PDS composites should be used only for defects smaller than 1–2 cm^2^ [10]. Titanium mesh offers higher strength and stability, but may cause tenderness, weather sensations, cicatricial eye movement disorders and beam hardening in x-ray examinations [12, 13, 19]. UHMWPE is an innovative biomaterial, which seemingly combines the advantages of PDS and titanium mesh, making it a suitable material for the reconstruction of orbital floor fractures. The present study is the first to investigate, if the use of UHMWPE leads to sufficient reconstruction of orbital volumes compared to a conventional Polyglactin 910/PDS composite patch (Ethisorb, Ethicon, Johnson & Johnson, New Brunswick, NJ, USA). For this purpose, UHMWPE (marPOR, KLS Martin Group, Tuttlingen, Germany) with two different strengths, 0.85 mm and 1.5 mm, was used. All materials led to statistically significant restoration of intact orbital volumes (orbital volumes before the fracture), but orbits that were reconstructed using marPOR with a strength of 0.85 mm exhibited the most accurate restoration with an absolute volume difference of − 0.05 mL from the mean volume of intact orbits. Using marPOR with a strength of 1.5 mm led to slight overcompensation. Mean orbital volumes tended to be smaller than intact orbital volumes with an absolute mean volume difference of − 0.34 mL, probably due to the simple size of the material. Even though Ethisorb is the thinnest material with a strength of 0.5 mm, orbits that were reconstructed using Ethisorb turned out to be 0.21 mL smaller on average compared to mean intact orbital volumes. Despite its thickness of 0.85 mm, marPOR offers a high ductility. The properties of UHMWPE are highly dependent on its microstructure rather than molecular mass, resulting in a very stable, but light-weight material with a molecular weight of 3.5–7.5 million g/mole [20, 21]. Furthermore, it provides a very high modulus of elasticity with 0.5–0.8 GPa. With its three-dimensional, highly porous compound structure, marPOR provides best conditions for neovascularization, osseointegration and therefore a good long-term stability [21]. Due to the porous structure, potential retrobulbar hemorrhage should not lead to any increase in retrobulbar pressure. The results and properties implicate that UHMWPE (marPOR) is not only very stable, preventing any soft tissue from prolapsing through the fracture, but also gently adapts to the bony contour of the orbit, which in turn leads to the most accurate volume restoration. In this context, it provides good manual ductility during the surgery. On the other hand, the stability of high-weight titanium mesh comes at the price of worse flexibility, demanding pre-contoured patient-specific titanium mesh for adequate orbital reconstruction, which is costly and time intensive, respectively [22]. In contrast to titanium mesh, UHMWPE is also radiolucent, does not affect any x-ray examinations and should not lead to tenderness or weather sensations [23]. Moreover, UHMWPE’s significance for achieving outstanding performances in total joint arthroplasties is unquestionably proven not only by its high wear-resistance, biocompatibility, durability, ductility and toughness, but also by the low infection rates below 1% of patients. [21, 24]. This may be a huge advantage over PDS as well as Polyglactin 910/PDS composites with reported infection rates up to 4% [7, 8]. However, further studies must clarify if this is also applicable in the context of orbital floor reconstruction. Because Polyglactin 910 and PDS are biodegradable materials, long-term stability, remnant defects and patient outcome significantly rely on the plates’ resorption rate. The risk of remnant defects have been increased as the plates had incomplete resorption, affecting one third of patients in a study of Tabrizi et al. [25]. This should not be seen in orbits reconstructed with marPOR as it is an inert, non-biodegradable material. We conclude that marPOR is an appropriate alternative material to be used for the reconstruction of orbital floor defects. However, clinical studies must be conducted in order to prove the effectiveness and safeness of marPOR in the context of orbital floor reconstruction. As it is already an approved CE medical product, marPOR could be used for orbital floor reconstruction today, minimizing any obstacles for further clinical evaluation.
